# Illumina MiSeq Sequencing Reveals Diverse Microbial Communities of Activated Sludge Systems Stimulated by Different Aromatics for Indigo Biosynthesis from Indole

**DOI:** 10.1371/journal.pone.0125732

**Published:** 2015-04-30

**Authors:** Xuwang Zhang, Yuanyuan Qu, Qiao Ma, Zhaojing Zhang, Duanxing Li, Jingwei Wang, Wenli Shen, E Shen, Jiti Zhou

**Affiliations:** Key Laboratory of Industrial Ecology and Environmental Engineering (Ministry of Education), School of Environmental Science and Technology, Dalian University of Technology, Dalian, China; Wilfrid Laurier University, CANADA

## Abstract

Indole, as a typical N-heteroaromatic compound existed in coking wastewater, can be used for bio-indigo production. The microbial production of indigo from indole has been widely reported during the last decades using culture-dependent methods, but few studies have been carried out by microbial communities. Herein, three activated sludge systems stimulated by different aromatics, i.e. naphthalene plus indole (G1), phenol plus indole (G2) and indole only (G3), were constructed for indigo production from indole. During the operation, G1 produced the highest indigo yield in the early stage, but it switched to G3 in the late stage. Based on LC-MS analysis, indigo was the major product in G1 and G3, while the purple product 2-(7-oxo-1H-indol-6(7H)-ylidene) indolin-3-one was dominant in G2. Illumina MiSeq sequencing of 16S rRNA gene amplicons was applied to analyze the microbial community structure and composition. Detrended correspondence analysis (DCA) and dissimilarity tests showed that the overall community structures of three groups changed significantly during the operation (*P*<0.05). Nevertheless, the bacteria assigned to phylum *Proteobacteria*, family *Comamonadaceae*, and genera *Diaphorobacter*, *Comamonas* and *Aquamicrobium* were commonly shared dominant populations. Pearson correlations were calculated to discern the relationship between microbial communities and indigo yields. The typical indigo-producing populations *Comamonas* and *Pseudomonas* showed no positive correlations with indigo yields, while there emerged many other genera that exhibited positive relationships, such as *Aquamicrobium*, *Truepera* and *Pusillimonas*, which had not been reported for indigo production previously. The present study should provide new insights into indigo bio-production by microbial communities from indole.

## Introduction

Coking wastewater is a typical industrial wastewater, usually containing high levels of inorganic pollutants, phenolic compounds, polynuclear aromatic hydrocarbons and N-heteroaromatic compounds. [1,2]. Indole is one of the typical N-heteroaromatics in coking wastewater, which is generally degraded into H_2_O, CO_2_ (aerobic) or CH_4_ (anaerobic) by microbial process [1]. In recent decades, the production of specific chemicals (e.g. bio-hydrogen, bioplastics and organic solvents) from waste streams by microbiome has gained great interests owing to the increasing demand for sustainable resources of chemicals and fuels [3,4]. Likewise, indole can be used as raw material for microbial production of indigo, which is one of the oldest dyestuffs widely used in textile industry for the dyeing of cotton and denim fabrics [5–9].

During the past 30 years, various bacterial strains have been isolated with the capability of producing indigo from indole, and most of them belong to *Pseudomonas* [5,6,10], *Acinetobacter* [7,11] and *Comamonas* [8]. Indole can be oxidized by varieties of mono- or di-oxygenases in those bacterial strains to form indoxyl, which spontaneously dimerizes to form indigo [12–14]. The expression of those oxygenases can be stimulated by different aromatics, such as naphthalene (naphthalene dioxygenase), phenol (phenol hydroxylase) and styrene (styrene monooxygenase) [5–7]. Besides, indole can also stimulate the expression of monooxygenase or dioxygenase for the production of indigo in some bacterial strains [11,15]. Although many oxygenases can catalyze the process of indigo production, the capabilities of the indigo-producing strains are different, resulting in the varying yields of indigo [6,7]. In addition, various by-products are also produced, including isatin, indirubin and 7-hydroxyindole, and some products can serve as the chief precursors for production of dyes and pharmaceuticals [14,16–18]. The production of various indigoids is probably owing to the regioselectivity of the enzymes stimulated by different aromatics.

While microbial production of indigo has been intensively studied by culture-dependent methods [6,8,10,12], surprisingly limited research has been conducted to explore the possibility of microbial community for indigo production. Compared with pure cultures, microbial community can be more robust and stable to environmental fluctuation, which is preferred to be used for the synthesis of fine chemicals from waste [3,4]. Recently, many studies have been done to investigate the dynamics and diversity of microbial communities from different wastewater treatment systems by high-throughput sequencing, which enables us to detect massive microbial populations at a high throughput and low cost way [19–24]. A better understanding of microbial communities for indigo production will not only expand our knowledge of microbial indigo production, but also provide useful information for indole containing wastewater treatment.

In this study, the activated sludge (AS) stimulated by different aromatics was used to produce indigo from indole. Naphthalene and phenol, as two typical aromatics existed in coking wastewater, were selected as the stimulating substrates. With the aid of Illumina MiSeq sequencing technology, an attempt was made to characterize and compare the bacterial communities under different conditions. Additionally, correlations of microbial communities in the relative abundance with indigo yields were performed to reveal the possible populations responsible for indigo production in different activated sludge systems.

## Materials and Methods

### Experimental setup and operation conditions

Three sequencing batch reactors (SBRs) were simulated with 250-mL flasks containing 100 mL synthetic wastewater, which consisted of 6 g/L Na_2_HPO_4_, 3 g/L KH_2_PO_4_, 0.5 g/L NaCl, 1 g/L NH_4_Cl, 0.011 g/L CaCl_2_, and 0.24 g/L MgSO_4_. At the beginning, the three SBRs were seeded with the activated sludge (0.54 g, dry weight at 105°C) from Chunliu River Wastewater Treatment Plant (Dalian, China) and domesticated with 200 mg/L naphthalene (G1), 200 mg/L phenol (G2), and 100 mg/L indole (G3), respectively. Each of the group contained one SBR. After 15 days, the three SBRs were operated in parallel over 69 days with the influent containing naphthalene plus indole (G1), phenol plus indole (G2), and indole only (G3), respectively. During the operation, both naphthalene and phenol were maintained at an appropriate concentration of 200 mg/L, while the concentration of indole was increased gradually from 72–88 to 168–174 mg/L. Each operation cycle of SBR was operated for 72 h, including 2 h filling, 66 h reacting, 2 h settling, and 2 h decanting. Samples were taken at the end of each SBR operation cycle to measure the yields of indigo and the residual concentrations of indole by high performance liquid chromatography (HPLC) (Shimadzu LC20A, Japan; Thermo Hypersil ODS-2 column, 5 μm, 250×4.6 mm). The pigments produced in three reactors were also analyzed by HPLC-mass spectroscopy (MS). HPLC and MS were performed as described previously [8,9].

### DNA extraction, PCR and sequencing

In order to comprehensively analyze the microbial communities during the operation, 11 activated sludge samples were collected from each SBR for Illumina MiSeq sequencing, among which five were taken within 0–20 days, three within 20–40 days, and three within 40–69 days (Table A in S1 File). Samples were taken concurrently from the three groups. The genomic DNA was extracted using the protocol reported previously [25], and the DNA concentration was determined by Pico Green assay using a FLUOstar OPTIMA fluorescence plate reader (BMG Labtech, Germany). PCR amplification was carried out using the primer set 515F (5’-GTG CCA GCM GCC GCG GTA A-3’) and 806R (5’-GGA CTA CHV GGG TWT CTA AT-3’) for the V4 region of 16S rRNA gene. The 25 μL PCR mixture contained 0.1 μL of AccuPrime High Fidelity Taq Polymerase, 1 μL of each primer (10 μM), 2.5 μL of 10×AccuPrime PCR buffer II (Invitrogen, USA), and 1 μL of template DNA. PCR was performed in a Veriti 96-Well Thermal Cycler (Applied Biosystems, USA) under the following thermocycling steps: initial pre-denaturation at 94°C for 1 min, followed by 35 cycles of denaturation at 94°C for 20 s, annealing at 53°C for 25 s, and elongation at 68°C for 45 s, with a final extension at 68°C for 10 min. Each sample was amplified in triplicate. The resulting PCR products were pooled and purified using QIAquick Gel Extraction Kit (Qiagen, Germany). After purification, the 16S rRNA V4 region PCR products were quantified by Pico Green analysis. A mixture of the amplicons was then used for sequencing on Illumina MiSeq platform at the Institute for Environmental Genomics, University of Oklahoma. During the sequencing, sterile water samples in place of DNA were used as the negative controls to eliminate the influence of contaminating DNA, and the background data were subtracted.

### Data analysis

After sequencing, the primers and spacers were trimmed. The paired-end (PE) reads were overlapped to assemble the V4 tag sequences using the Flash program [26]. To minimize the effects of random sequencing error, both the low quality fragments and the sequences shorter than 240 bp were removed. The PCR chimeras were checked and filtered out by UCHIME [27]. Each sample was randomly re-sampled and normalized at 25,230 sequences. The sequences were classified into operational taxonomic units (OTU) by setting a 0.03 distance limit using the CD-HIT program [28]. The taxonomic assignment of OTUs was performed by RDP classifier at 50% threshold [29]. The Shannon diversity index, Simpson diversity index and rarefaction curves were generated using Mothur program [30]. Detrended correspondence analysis (DCA) was conducted by Canoco 4.5. Three dissimilarity tests, i.e. multiple-response permutation procedure (MRPP), permutational multivariate analysis of variance (Adonis), and analysis of similarity (ANOSIM), were performed with Bray-Curtis distances using R environment (version 3.1.0; http://www.r-project.org/). Heat map analysis of the 10 most abundant genera in each group was conducted by R environment (version 3.1.0). Pairwise Pearson correlation was calculated to illuminate the relationship between microbial community compositions and indigo yields. The raw sequencing data have been submitted to NCBI Sequence Read Archive (http://www.ncbi.nlm.nih.gov/sra/) with the project accession number of SRP055799.

## Results and Discussion

### Process performance of indigo production

**Fig 1:**
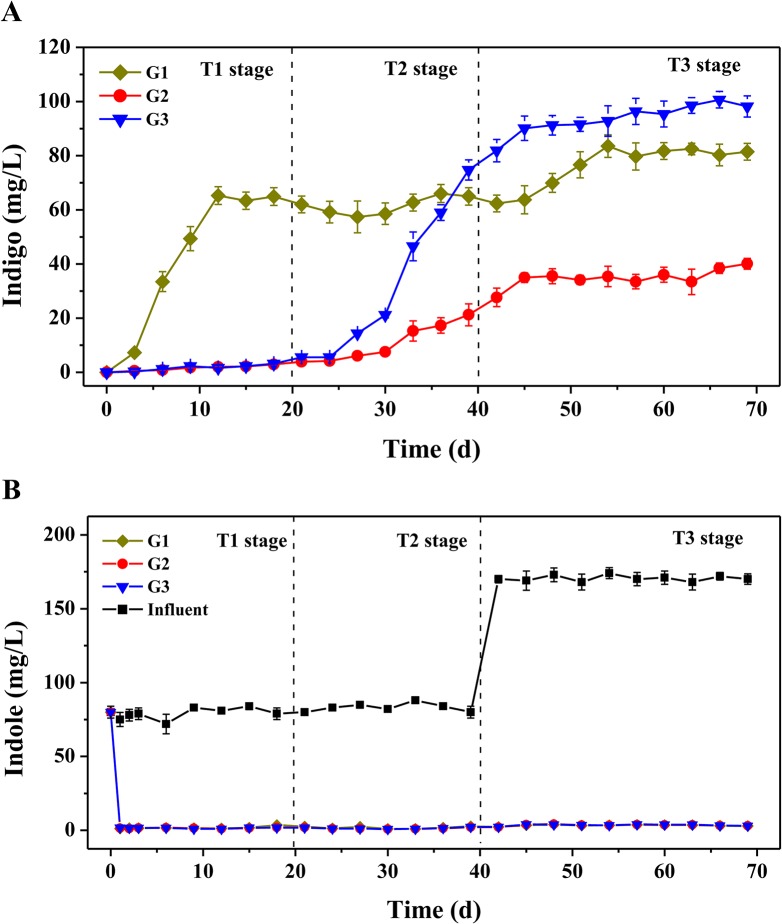
Biosynthesis of indigo from indole by three activated sludge systems stimulated by different aromatics. **A.** Indigo production by three activated sludge systems. **B.** Indole consumption by three activated sludge systems. G1, naphthalene plus indole; G2, phenol plus indole; G3, indole only. The concentrations of indigo and residual indole were measured by HPLC at the end of each SBR operation cycle. Detailed group setup were presented in Table A in S1 File.

Three activated sludge systems were constructed for indigo production from indole using different aromatics as the stimulating substrates (Table A in S1 File). Fig 1A depicts the indigo production performance over 69-day operation, which could be roughly divided into three stages, i.e. T1 (0–20 days), T2 (20–40 days) and T3 (40–69 days). During the initial 20 days (T1), G1 exhibited a better capability for indigo production, and the indigo yields became stable (~60 mg/L) in 12 days. Compared to G1, the indigo yields in G2 and G3 were almost negligible (less than 3 mg/L), which, thereafter, increased gradually in T2 stage, and G3 produced higher levels of indigo than G1 in T3 stage. At the end of operation, the indigo yields in three groups all reached the plateaus. G3 produced the highest yield of indigo (~97 mg/L), followed by G1 (~81 mg/L) and G2 (~36 mg/L). The results proved the possibility of producing indigo from indole by microbial communities, and the activated sludge system stimulated by indole unexpectedly produced the highest yields of indigo. During the operation, indole was almost completely removed in all three groups within each SBR operation cycle (Fig 1B), indicating the high efficiency of the activated sludge systems for the treatment of indole. Meanwhile, almost no naphthalene and phenol were detected by HPLC in the effluents of G1 and G2, respectively, suggesting both aromatics could be completed consumed by the sludge microbial communities.

The color of the pigments extracted in dimethylsulfoxide showed remarkable differences among three groups (Fig A in S1 File), which indicated that the compositions of the products could be different. To further identify the products, HPLC-MS analysis was performed. Two major products with the same prominent molecular ion (MH^+^) peak of m/z 263 were detected (Fig B in S1 File). The one (product I) with the retention time of 7.3 min was confirmed to be indigo, which had the same UV-vis spectrum and the fragmentation patterns with the commercial indigo standard. As for the other one (product II), the UV-vis spectrum was detected with absorption maxima at wavelengths of 221, 266, 314, 588 nm (Fig B in S1 File). In our previous study, a purple product, 2-(7-oxo-1H-indol-6(7H)-ylidene) indolin-3-one, was produced from indole by phenol hydroxylase [14], which had the similar UV-vis spectrum and the fragmentation patterns with the product II. Therefore, product II with the retention time of 11.3 min might be 2-(7-oxo-1H-indol-6(7H)-ylidene) indolin-3-one. Furthermore, the purple product in G2 might have a high relative abundance (Fig B in S1 File), resulting in the dark blue color of the products in G2 (Fig A in S1 File). It could be presumed that the enzyme responsible for indigoids production in G2 might be phenol hydroxylase, while the functional enzyme in G1 could be naphthalene dioxygenase. Both oxygenases can hydroxylate indole to form indoxyl, which may be further oxidized to form isatin (Fig C in S1 File) [8,14]. Condensation of two molecules of indoxyl by air oxidation will lead to the production of indigo (Fig C in S1 File) [12–14]. Besides, phenol hydroxylase can also oxidize indole through C-7 oxidation pathway to form 7-hydroxyindole, which can react with isatin to form 2-(7-oxo-1H-indol-6(7H)-ylidene) indolin-3-one (Fig C in S1 File) [14]. Previous studies demonstrated that both phenol hydroxylase and naphthalene dioxygenase could catalyze the production of dyestuffs from indole, but the products were not exactly the same [9,14,16,17]. As for G3, although indigo was the major compound, a small amount of purple product was also produced, and fewer by-products were produced compared to G1 (Fig B in S1 File). Therefore, the enzymes in G3 might be different from those in G1 and G2. Many oxygenases, such as P450 enzymes, flavin-containing monooxygenases and arene di/mono-oxygenase, have been proved to be capable of oxidizing indole into hydroxyindoles, which can result in indigoids formation under aerobic conditions [31,32]. On the other hand, the functional enzymes, catalyzing the first step of indole oxidation, in indole-degrading bacteria and microbial communities have not been identified and characterized up to now, which may also lead to indigoids production. Related researches are being conducted in our lab for gaining more insights into indole transformation. In the present study, the differences in enzymes and indigo production might be due to the variety of microbial communities among the three groups.

### Overview of microbial community diversity

Using Illumina MiSeq sequencing, a set of more than 25,000 effective sequence tags were yielded for each sample with an average length of 253 bp, resulting in a total of 907,207 sequences. Tags with 97% similarity were then grouped into OTUs by CD-HIT clustering method, and 2110 (G1), 2480 (G2) and 2168 (G3) OTUs were obtained (Table B in S1 File). However, the rarefaction curves did not approach saturation (Fig D in S1 File), indicating that there might be some microbes still remained undetermined. The total number of OTUs estimated by Chao1 estimator were 2752 (G1), 3188 (G2) and 2740 (G3) with infinite sampling (Table B in S1 File), suggesting G2 exhibited greater richness than G1 and G3 [22]. To assess the diversity and evenness of microbial populations among three groups, Shannon index (H) and Simpson index (D) were calculated. The higher the value of H, the richer the diversity; while the higher the D index, the lower the diversity [22,23,33]. Results showed that G2 had the highest diversity among three communities (Table B in S1 File). In previous studies, it was found that the chemicals with more number of rings exerted greater toxicity [34], and the impairment increased with increase in nitrogen content within the ring [35]. Therefore, phenol (one ring) might be less toxic than naphthalene (two rings) and indole (N-heterocyclic compound), thus leading to the less harmful effects on microbial communities. The original AS had a bigger H index and a smaller D index compared to three groups (Table B in S1 File), indicating that the addition of the aromatics would reduce the α-diversity of microbial community.

### Dynamic shift of microbial communities during the operation

**Fig 2:**
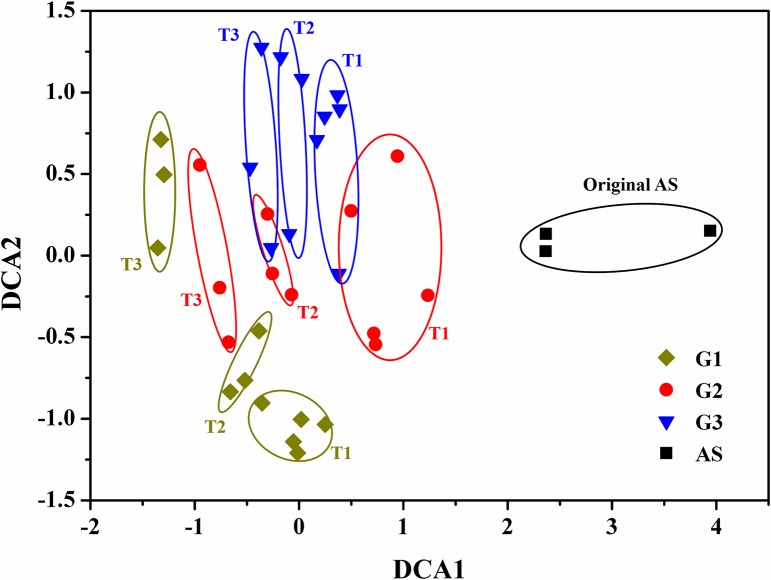
Detrended correspondence analysis of bacterial communities from three groups based on Illumina MiSeq sequencing. Detailed group setup were presented in Table A in S1 File. T1: 0–20 days; T2: 20–40 days; T3: 40–69 days.

DCA was used to examine the overall variation of the bacterial communities among three groups (Fig 2). The results revealed the whole process could be also divided into three stages, corresponding to three phases of indigo production (Fig 1). In T1 stage, the samples from three groups were all separated from the original activated sludge, indicating that the added aromatics would significantly affect the bacterial community structures. Within three groups, the samples from G2 and G3 were clustered together, which were well separated from that of G1. The results were consistent with the indigo production. In T2 stage, the samples from G2 and G3 shifted into two clusters, which were still separated from G1. In this stage, the indigo yields were improved to different extents in G2 and G3, while G1 maintained stable (Fig 1). Then, the samples diverged into three distinct subgroups in T3 stage, and the indigo production reached relative stability in three groups. In addition, according to three dissimilarity tests, i.e. MRPP, Adonis and ANOSIM, the community structures among three stages were significantly different (*P*<0.05) (Table C in S1 File) [36,37]. Similarly, the community structures among three groups were also significantly different with all three tests (*P*<0.05) (Table C in S1 File). These results suggested that there was a noticeable shift in community structures among three groups from the beginning of operation to the end.

Previous studies demonstrated that both deterministic and stochastic processes could be involved in shaping the communities during the operation [36–38]. Since the aromatics (naphthalene, phenol and indole) were served as the sole carbon source in influent, the communities could be highly stressed, thus deterministic processes would play significant roles in community assembly due to the selective pressure imposed by different aromatics, leading to differences of community structure among three groups [37]. In the meantime, stochastic processes, such as birth, death, colonization and extinction, could occur simultaneously during the operation, which would also affect the assembly of bacterial communities [36–38]. Especially, when the communities were stimulated gradually, the differences of community structure caused by deterministic and stochastic processes would be enhanced due to the competitive interactions [37], leading to the dissimilarity in indigo production among three groups.

### Bacterial community structures of three groups

**Fig 3:**
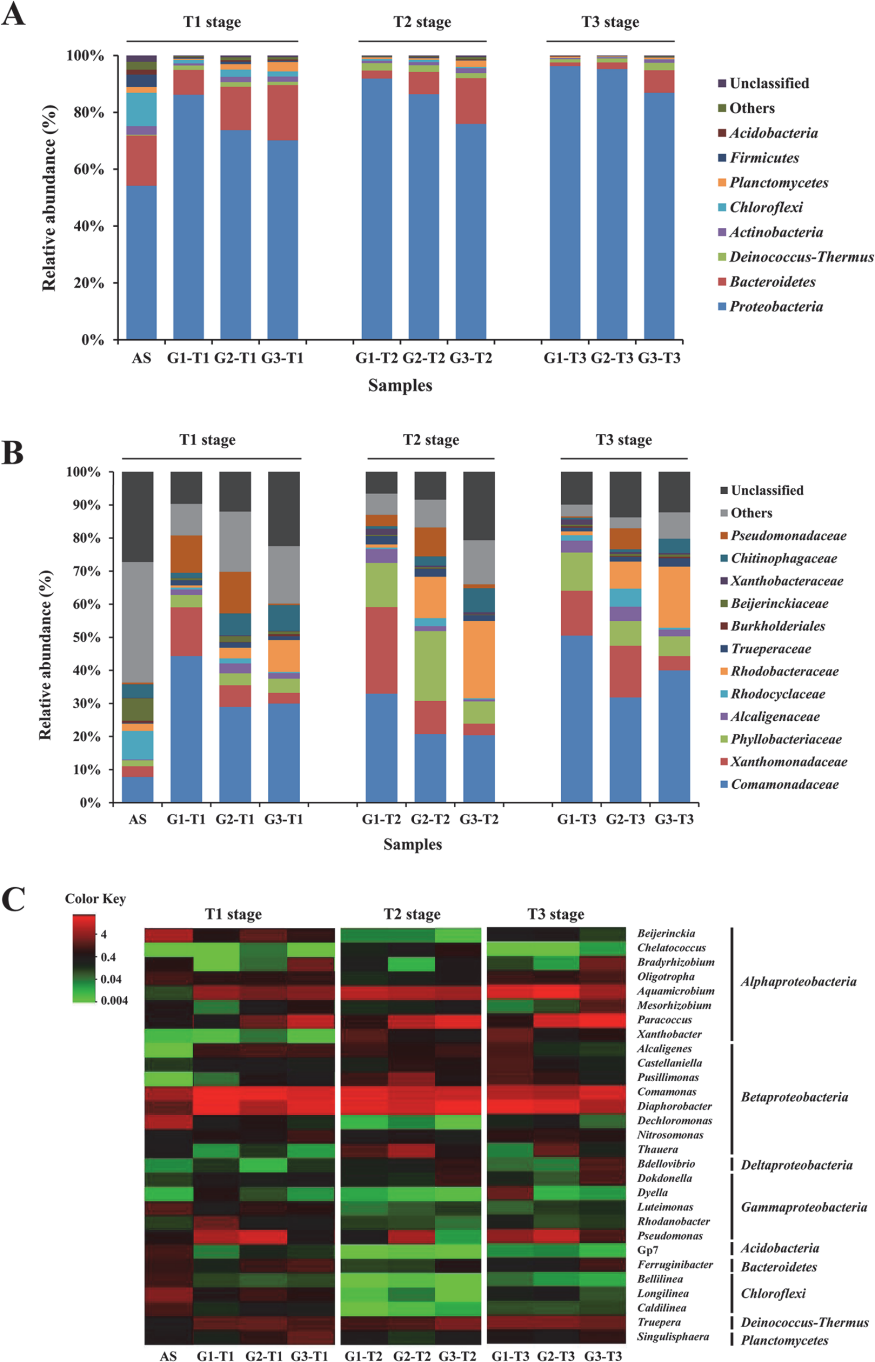
Taxonomic classification of sequences from bacterial communities of the three groups. **A.** Relative abundance of the dominant phyla in each group at different stages. **B.** Relative abundance of the dominant families in each group at different stages. **C.** Heat map of the 10 most abundant genera in each group at different stages. The color intensity in each cell showed the abundance of a genus in a group. RDP classifier was used to assign the sequences to different taxonomic levels at 50% threshold, and the abundances were displayed as percentage of the total sequences in each group.

To identify the taxonomic diversity of bacterial communities among three groups, the RDP classifier was used to assign the sequence tags to different taxonomic levels (from phylum to genus) at 50% threshold. The three communities harbored diverse lineages of bacterial phyla, which were reflected by the fact that 24 (G1), 24 (G2) and 26 (G3) bacterial phyla were detected. As shown in Fig 3A, *Proteobacteria* was the predominant phylum in all samples, accounting for 70–96% of total effective bacterial sequences. Similar results were present in previous studies, which found that *Proteobacteria* was the dominant community in soil [39], sediments [33], and wastewater treatment plants [21,40]. Other dominant phyla were *Bacteroidetes* (1.3–19.5%), *Deinococcus-Thermus* (1.1–2.6%), and *Actinobacteria* (0.4–1.8%). These four phyla accounted for approximately 92–99% of the classified sequences within three groups. Whilst, the microbial communities of the original AS were much more diverse, with a preponderance of *Proteobacteria* (54.2%), *Bacteroidetes* (17.7%), *Chloroflexi* (11.7%), *Firmicutes* (4.3%), *Actinobacteria* (3.1%), *Planctomycetes* (2.0%) and *Acidobacteria* (1.7%).

Within *Proteobacteria*, *Betaproteobacteria* was the most dominant group (21–65%) (Fig E in S1 File). This finding was similar to the results of previous studies, which revealed that the *Beta*-subdivision was the predominant one within *Proteobacteria* in the activated sludge samples [21,40]. Other studies reported different observations, where *Alphaproteobacteria* was the primary subdivision of *Proteobacteria* in the samples of wastewater treatment systems [20]. This disparity could be related to the differences in wastewater characteristics and treatment processes. The other main subgroups of identifiable sequences were related to *Alphaproteobacteria* (8.2–38.9%), *Gammaproteobacteria* (4.6–29.9%) and *Deltaproteobacteria* (0.2–2.8%), and the abundances of the unclassified *Proteobacteria* were less than 1%. In G1, *Betaproteobacteria* became more abundant than that in G2 and G3, while *Alphaproteobacteria* was less abundant. However, the abundance of *Gammaproteobacteria* in G3 was quite lower than those in G1 and G2 throughout the process, suggesting that indole might be more harmful to *Gammaproteobacteria* than naphthalene and phenol.

At the family level, a total of 156 families were obtained, and the majority of sequences belonged to 10 families (>1% on average) (Fig 3B). *Comamonadaceae* was frequently detected with the highest abundances in almost all samples. Compared to the original AS, *Rhodocyclaceae* and *Beijerinckiaceae* were less abundant in three groups, but the abundance of *Comamonadaceae* increased significantly, along with *Xanthomonadaceae*, *Phyllobacteriaceae* and *Alcaligenaceae*. In G3, the families *Rhodobacteraceae* and *Chitinophagaceae* were highly represented compared to G1 and G2, but *Xanthomonadaceae*, *Rhodocyclaceae* and *Pseudomonadaceae* had a rather lower abundance.

The heat map showed the profiles of the 10 most abundant genera in each group at different stages, and a total of 29 genera were selected for comparison (Fig 3C). Ten genera were abundant (>1%) in the original activated sludge with *Dechloromonas* (6.2%) and *Beijerinckia* (5.8%) being the most dominant. *Dechloromonas* has been extensively reported as the typical bacteria in activated sludge [21], while *Beijerinckia* has been considered as dinitrogen-fixing bacterium that could utilize a variety of multicarbon compounds [41]. But the abundances of both genera decreased significantly after the aromatics were added. Among the top 10 genera in three groups, *Diaphorobacter*, *Comamonas* and *Aquamicrobium* were commonly shared with relatively high abundances (>1%), which were previously reported with the ability of aromatics-degrading [42–44]. *Truepera* increased significantly, which was the only dominant genus (>1%) belonging to phylum *Deinococcus-Thermus* other than *Proteobacteria*. *Pseudomonas*, as the common genus for phenol degradation [45], existed in high abundances (6.3–12.5%) in G2, but in low abundances (0.02–1.1%) in G3.

### Correlations of microbial communities and indigo production

To discern the possible relationship between microbial community compositions and indigo production, the Pearson correlation test was performed at each level. As shown in Table 1, it could be observed that the majority of the community compositions were not significantly correlated to indigo yields, and more groups at different taxonomic levels showed negative relationships rather than positive.

**Table 1:** The number of microbial community composition showing significant correlation between the relative abundance and indigo yields in each group at different taxonomic levels.

Taxon	G1			G2			G3		
	P^a^	N^b^	None^c^	P	N	None	P	N	None
**Phylum**	1	11	10	1	9	13	3	9	13
**Family**	3	36	84	4	33	97	5	35	88
**Genus**	3	59	218	12	61	234	15	69	202

^a^P: positive relationship (r>0, *P*<0.05).

^b^N: negative relationship (r<0, *P*<0.05).

^c^None: no significant relationship (*P*>0.05).

**Fig 4:**
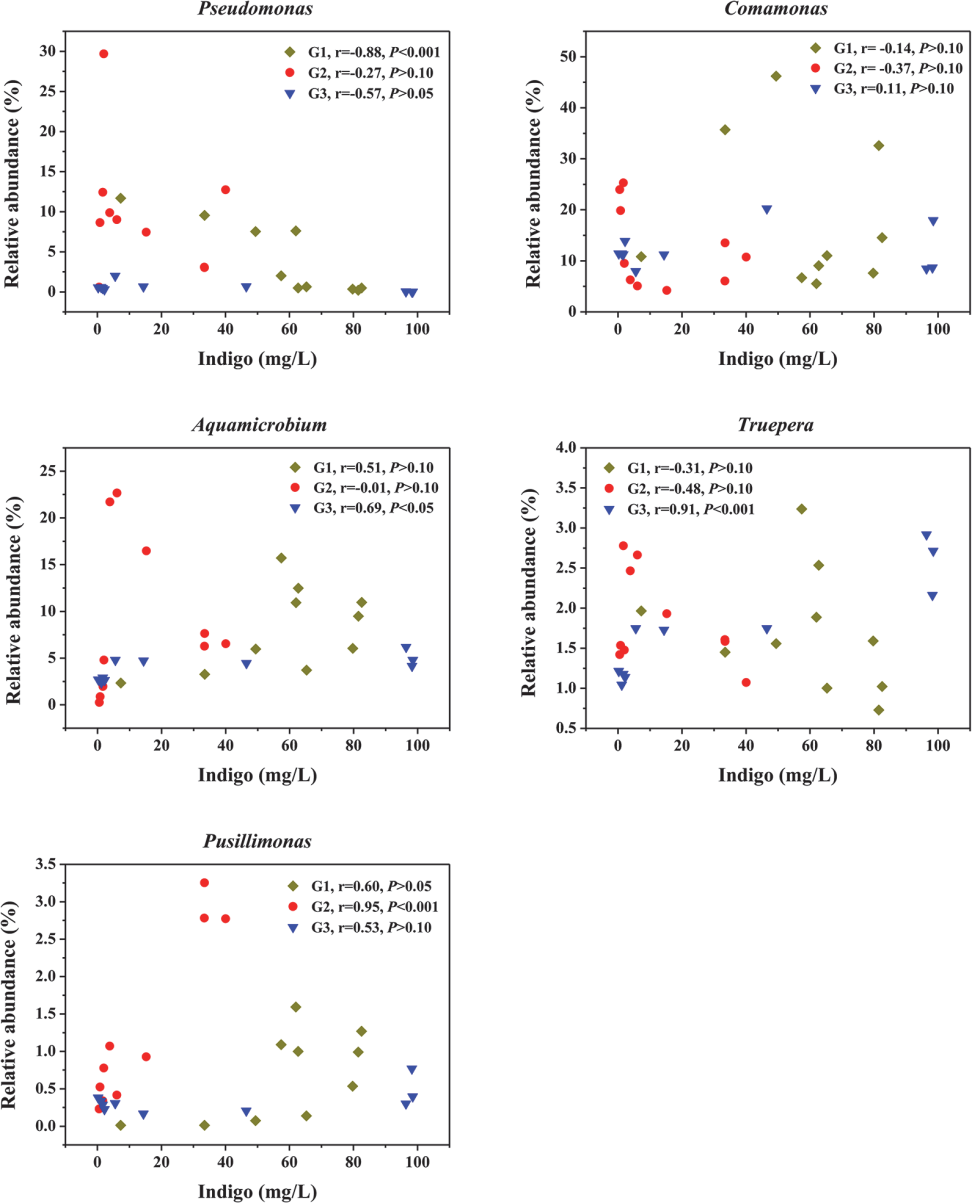
Correlations between the relative abundance of different genera and indigo yields. Pearson correlation coefficients (r) with the associated *P* values were shown for each genus of each group.

Among the major genera, the relative abundances of *Comamonas*, *Diaphorobacter* and *Paracoccus* had no significant correlation with indigo yields in all three groups (*P*>0.05) (Table D in S1 File, Fig 4). *Pseudomonas* decreased in relative abundance as indigo yields increased in G1 (*P*<0.001), while the relative abundance of *Pusillimonas* increased with indigo yields in G2 (*P*<0.001) (Fig 4). The relative abundances of *Aquamicrobium* and *Truepera* were both positively correlated with indigo yields in G3 (*P*<0.05) (Fig 4). Other significantly correlated genera included *Castellaniella* (positive in G2 and G3), *Mesorhizobium* (positive in G1), *Brevundimonas* (positive in G2 and G3), *Sphingosinicella* (positive in G2 and G3), *Longilinea* (negative in all three groups), *Dechloromonas* (negative in G2 and G3) and *Ferruginibacter* (negative in G3) (Table D in S1 File), but each genus accounted for <1% of the total sequences.

To determine the relationship between the unclassified sequences and indigo yields, six major unclassified OTUs, which accounted for >1% of the total sequences in more than two groups, were selected for correlation analysis. Four OTUs belonged to phylum *Proteobacteria*, among which the relative abundances of OTU_2732 and OTU_3338 showed no significant correlation with indigo yields in all three groups (*P*>0.05) while the abundances of OTU_129 and OTU_863 were significantly positively correlated to indigo yields in G3 and G2, respectively (*P*<0.05) (Table E in S1 File). On the contrary, the other two OTUs, belonged to phylum *Bacteroidetes*, showed negative correlations with indigo yields (Table E in S1 File). Since *Proteobacteria*, as the most abundant phylum, showed significantly positive correlation between the abundances and indigo yields in all three groups (Table D in S1 File), those rare or unclassified sequences might act as potential contributing factors.

According to the previous studies, a wide phylogenetic diversity of bacteria were capable of producing indigo from indole, including genera *Acinetobacter* [7,11], *Comamonas* [8], *Pseudomonas* [5,6,10], *Rhodococcus* [46] and *Sphingomonas* [47]. Among them, *Pseudomonas* spp. have been most extensively studied. Herein, though genera *Comamonas* and *Pseudomonas* occupied considerable abundances in three communities, they might not play the key role in indigo production based on the Pearson correlation test, and *Pseudomonas* even exhibited negative effects. Whilst, other typical indigo-producing bacterial strains were rarely existed in the communities (less than 0.01% of the total sequences). Therefore, there might be some novel strains favoring in indigo production which had not been reported previously, e.g., *Aquamicrobium*, *Truepera* and *Pusillimonas*. In previous studies, *Aquamicrobium* exhibited a good ability for the degradation of biphenyl and polychlorinated biphenyls [43], *Truepera* was reported to be resistant to ionizing radiation [48], and *Pusillimonas* was capable of alkanes degradation [49]. Owing to the significant positive correlations, those strains were probably served as new biocatalysts for indigo production.

## Conclusions

In this study, the microbial communities were proved to be capable of producing indigo from indole, but the indigo production in the activated sludge systems stimulated by different aromatics was dissimilar. Illumina MiSeq sequencing showed that three communities changed significantly during the operation. Pearson correlation tests suggested that there should be more bacteria with the capability of producing indigo from indole remaining unreported. This work should provide important information for a more comprehensive understanding of microbial indigo production.

## Supporting Information

S1 File This includes Tables A-E and Figures A-E. Table A. Group setup of the experiment in this study. Table B. Richness and diversity indices of the original AS and three groups. Table C. Significance tests of the differences of the microbial communities. Table D. Correlations between the relative abundance of microbial community compositions and indigo yields. Table E. Correlations between the relative abundance of the major unclassified OTU and indigo yields. Fig A. Color of the products produced by the three groups. The reaction mixture were centrifuged at 10,000×*g* for 5 min, and the pellets were re-suspended in an equal volume of dimethylsulfoxide for the extraction of indigoid pigments. Fig B. HPLC-MS analysis of the products formed by the three groups. a. HPLC spectra of the products. b. Mass spectra of the product indigo. c. Mass spectra of the purple product. The pigments of I and II were indicated by the arrows, corresponding to indigo and purple product, 2-(7-oxo-1H-indol-6(7H)-ylidene) indolin-3-one, respectively. Fig C. Proposed pathways for the production of indigoids from indole by the activated sludge systems. Indole can be hydroxylated to form indoxyl by oxygenases A and B, which may be further oxidized to form isatin. Condensation of two molecules of indoxyl by air oxidation will lead to the production of indigo. Besides, indole can be also oxidized by oxygenase B to form 7-hydroxyindole, which can react with isatin to form 2-(7-oxo-1H-indol-6(7H)-ylidene) indolin-3-one. Oxygenase A may be naphthalene dioxygenase in G1; B may be phenol hydrolase in G2 or other oxygeases in G3. Fig D. Rarefaction curves base on Illumina MiSeq sequencing of microbial communities. The OTUs were defined by 3% distances. The rarefaction curve of AS was derived from 3 samples, while those of each group were from 11 samples. The detailed group setup was presented in Table A in S1 File. Fig E. Relative abundance of *Proteobacteria* subdivisions in each group at different stages.
(PDF)
