# Supplementary material for: Illumina MiSeq Sequencing Reveals Diverse Microbial Communities of Activated Sludge Systems Stimulated by Different Aromatics for Indigo Biosynthesis from Indole
Source: PLoS One. 2015 Apr 30;10(4):e0125732. doi: 10.1371/journal.pone.0125732 (PMC4416020; doi:10.1371/journal.pone.0125732)
Supplement: S1 File — Table A. Group setup of the experiment in this study. Table B. Richness and diversity indices of the original AS and three groups. Table C. Significance tests of the differences of the microbial communities. Table D. Correlations between the relative abundance of microbial community compositions and indigo yields. Table E. Correlations between the relative abundance of the major unclassified OTU and indigo yields. Fig A. Color of the products produced by the three groups. The reaction mixture were centrifuged at 10,000×g for 5 min, and the pellets were re-suspended in an equal volume of dimethylsulfoxide for the extraction of indigoid pigments. Fig B. HPLC-MS analysis of the products formed by the three groups. a. HPLC spectra of the products. b. Mass spectra of the product indigo. c. Mass spectra of the purple product. The pigments of I and II were indicated by the arrows, corresponding to indigo and purple product, 2-(7-oxo-1H-indol-6(7H)-ylidene) indolin-3-one, respectively. Fig C. Proposed pathways for the production of indigoids from indole by the activated sludge systems. Indole can be hydroxylated to form indoxyl by oxygenases A and B, which may be further oxidized to form isatin. Condensation of two molecules of indoxyl by air oxidation will lead to the production of indigo. Besides, indole can be also oxidized by oxygenase B to form 7-hydroxyindole, which can react with isatin to form 2-(7-oxo-1H-indol-6(7H)-ylidene) indolin-3-one. Oxygenase A may be naphthalene dioxygenase in G1; B may be phenol hydrolase in G2 or other oxygeases in G3. Fig D. Rarefaction curves base on Illumina MiSeq sequencing of microbial communities. The OTUs were defined by 3% distances. The rarefaction curve of AS was derived from 3 samples, while those of each group were from 11 samples. The detailed group setup was presented in Table A in S1 File. Fig E. Relative abundance of Proteobacteria subdivisions in each group at different stages. (PDF) [file pone.0125732.s001.pdf]

Supporting Information for ***PLoS ONE***

Illumina MiSeq sequencing reveals diverse microbial communities of activated sludge systems stimulated by different aromatics for indigo biosynthesis from indole

Xuwang Zhang, Yuanyuan Qu*, Qiao Ma, Zhaojing Zhang, Duanxing Li, Jingwei Wang, Wenli Shen, E Shen, Jiti Zhou

Key Laboratory of Industrial Ecology and Environmental Engineering (Ministry of Education), School of Environmental Science and Technology, Dalian University of Technology, Dalian, China

*Corresponding Author

E-mail: qyy@dlut.edu.cn (YQ).

Table A Group setup of the experiment in this study.

Group	Time[a]	Samples	Conditions
AS	T0	A1, A2, A3	Original activated sludge
	T1	B1, B2, B3, B4, B5	
G1	T2	B6, B7, B8	Induced with naphthalene and indole
	T3	B9, B10, B11	
	T1	C1, C2, C3, C4, C5	
G2	T2	C6, C7, C8	Induced with phenol and indole
	T3	C9, C10, C11	
	T1	D1, D2, D3, D4, D5	
G3	T2	D6, D7, D8	Induced only with indole
	T3	D9, D10, D11	

[a]T0 stands for the operation period before domestication, and samples were selected from the original activated sludge; T1 stands for 0-20 days, and samples were selected at 0th, 3rd, 6th, 9th and 12th day; T2 stands for 20-40 days, and samples were selected at 21th, 27th and 33th day; T3 stands for 40-69 days, and samples were selected at 57th, 63th and 69th day.

Table B Richness and diversity indices of the original AS and three groups.

Groups	Shannon Index (H)[a]	Simpson Index (D)[b]	Chao1[c]	OTU
AS	5.90	0.011	2838	2553
G1	4.15	0.054	2752	2110
G2	4.74	0.025	3188	2480
G3	4.66	0.029	2740	2168

[a]Shannon index (H): a higher value represents more diversity.

[b]Simpson Index (D): a higher value represents less diversity.

[c]Chao1 richness estimator: a higher number indicates higher richness.

Table C Significance tests of the differences of the microbial communities[a].

Data sets	Adonis[b]		ANOSIM[c]		MRPP[d]	
	R^2	*P*	R	*P*	δ	*P*
T1 stage vs. T2 stage	0.108	0.008	0.169	0.031	0.632	0.006
T1 stage vs. T3 stage	0.173	0.001	0.437	0.001	0.633	<0.001
T2 stage vs. T3 stage	0.149	0.011	0.282	0.015	0.609	0.006
G1 vs. G2	0.164	0.001	0.366	0.001	0.631	<0.001
G1 vs. G3	0.254	0.001	0.606	0.001	0.576	<0.001
G2 vs. G3	0.189	0.001	0.517	0.001	0.581	<0.001

[a]Different statistical tests were used with Bray-Curtis distances, and *P* values were of corresponding significance tests.

[b]Adonis, permutational multivariate analysis of variance with the Adonis function.

[c]ANOSIM, analysis of similarity.

[d]MRPP, multiresponse permutation procedure.

Table D Correlations between the relative abundance of microbial community compositions and indigo yields.

Phylum	Class	Family	Genus	G1		G2		G3	
				r	*P* value[a]	r	*P* value	r	*P* value
Proteobacteria	-	-	-	**0.9890**	**<0.0001**	**0.8306**	**0.0029**	**0.9582**	**<0.0001**
Proteobacteria	*Betaproteobacteria*	*Comamonadaceae*	*Comamonas*	-0.1354	0.7092	-0.3654	0.2991	0.1118	0.7585
Proteobacteria	*Betaproteobacteria*	*Comamonadaceae*	*Diaphorobacter*	0.0703	0.8470	0.3487	0.3234	0.6063	0.0631
Proteobacteria	*Betaproteobacteria*	*Alcaligenaceae*	*Pusillimonas*	0.6029	0.0650	**0.9500**	**<0.0001**	0.5292	0.1157
Proteobacteria	*Alphaproteobacteria*	*Rhodobacteraceae*	*Paracoccus*	0.5497	0.0997	0.2645	0.4602	0.4012	0.2505
Proteobacteria	*Alphaproteobacteria*	*Phyllobacteriaceae*	*Aquamicrobium*	0.5110	0.1312	-0.0083	0.9818	**0.6862**	**0.0284**
Proteobacteria	*Gammaproteobacteria*	*Pseudomonadaceae*	*Pseudomonas*	**-0.8770**	**0.0009**	-0.2730	0.4454	-0.5692	0.0859
Deinococcus-Thermus	*Truepera*	*Trueperaceae*	*Truepera*	-0.3079	0.3868	-0.4780	0.1623	**0.9104**	**0.0003**
Proteobacteria	*Betaproteobacteria*	*Alcaligenaceae*	*Castellaniella*	-0.1272	0.7262	**0.7943**	**0.0061**	**0.8208**	**0.0036**
Proteobacteria	*Alphaproteobacteria*	*Phyllobacteriaceae*	*Mesorhizobium*	**0.7105**	**0.0213**	-0.1870	0.6049	-0.5030	0.1384
Proteobacteria	*Alphaproteobacteria*	*Caulobacteraceae*	*Brevundimonas*	-0.1522	0.6747	**0.9039**	**0.0003**	**0.9338**	**0.0001**
Proteobacteria	*Alphaproteobacteria*	*Sphingomonadaceae*	*Sphingosinicella*	0.6310	0.0504	**0.9379**	**0.0001**	**0.9736**	**<0.0001**
Proteobacteria	*Betaproteobacteria*	*Rhodocyclaceae*	*Dechloromonas*	-0.4604	0.1806	**-0.7929**	**0.0062**	**-0.7566**	**0.0113**
Chloroflexi	*Anaerolineae*	*Anaerolineaceae*	*Longilinea*	**-0.9557**	**<0.0001**	**-0.6476**	**0.0429**	**-0.7071**	**0.0222**
Bacteroidetes	*Sphingobacteria*	*Chitinophagaceae*	*Ferruginibacter*	-0.4813	0.1590	-0.5955	0.0693	**-0.9148**	**0.0002**

[a]$P<0.05$ indicated the correlation was significant.

Table E Correlations between the relative abundance of the major unclassified OTU and indigo yields.

OTU	Phylum	G1			G2			G3		
		Abundance[a]	r	*P* value[b]	Abundance	r	*P* value	Abundance	r	*P* value
OTU_129	*Proteobacteria*	13.50	0.1025	0.7781	5.53	0.5243	0.1198	1.27	**0.8813**	**0.0008**
OTU_2732	*Proteobacteria*	1.13	0.2982	0.4027	1.62	0.6669	0.0352	0.06	0.0996	0.7842
OTU_3338	*Proteobacteria*	0.80	0.4881	0.1523	1.67	0.5302	0.1149	2.29	-0.4118	0.2371
OTU_863	*Proteobacteria*	1.72	-0.1816	0.6157	0.56	**0.8933**	**0.0005**	4.40	0.2602	0.4678
OTU_300	*Bacteroidetes*	1.21	**-0.9208**	**0.0002**	3.09	**-0.7680**	**0.0095**	1.86	-0.5832	0.0768
OTU_473	*Bacteroidetes*	1.68	**-0.9703**	**0.0000**	0.08	**-0.8754**	**0.0009**	1.22	**-0.7791**	**0.0079**

[a]The average abundance of the OTU in each group was present in the table, and only the OTU with the abundance higher than 1% of the total sequences in more than two groups was selected.

[b]$P<0.05$ indicated the correlation was significant.

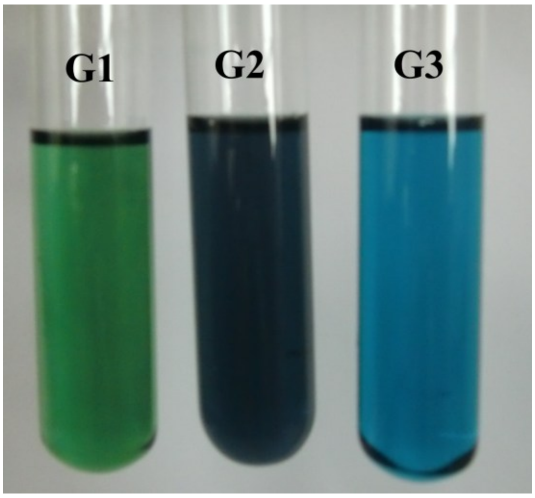

Fig A. Color of the products produced by the three groups. The reaction mixture were centrifuged at 10,000×*g* for 5 min, and the pellets were re-suspended in an equal volume of dimethylsulfoxide for the extraction of indigoid pigments.

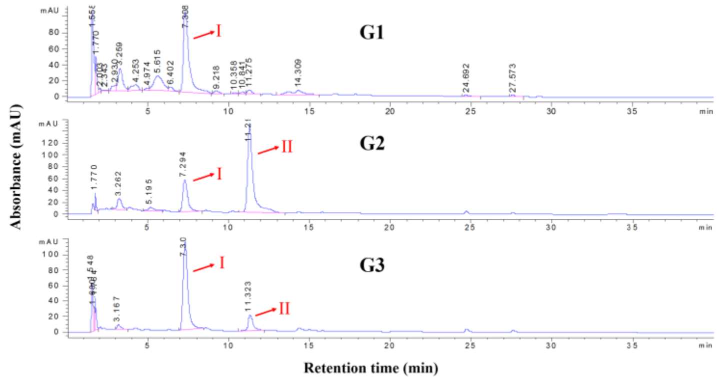

a

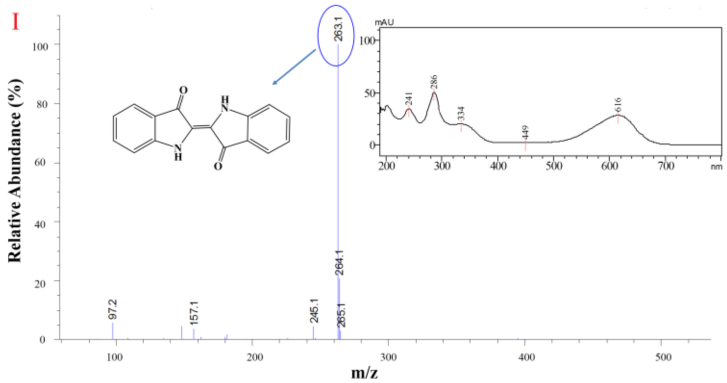

b

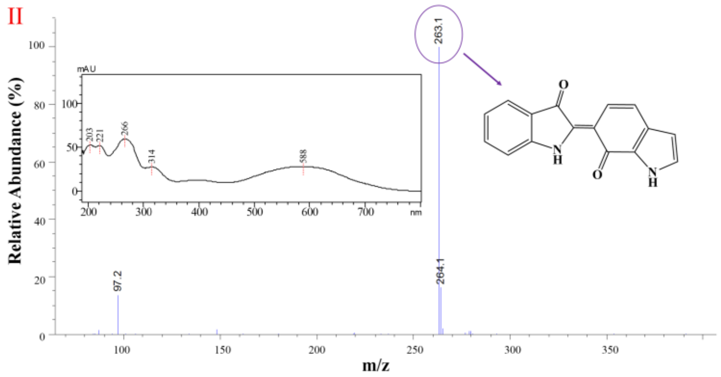

c

Fig B. HPLC-MS analysis of the products formed by the three groups. a. HPLC spectra of the products. **b.** Mass spectra of the product indigo. **c.** Mass spectra of the purple product. The pigments of I and II were indicated by the arrows, corresponding to indigo and purple product, 2-(7-oxo-1H-indol-6(7H)-ylidene) indolin-3-one, respectively.

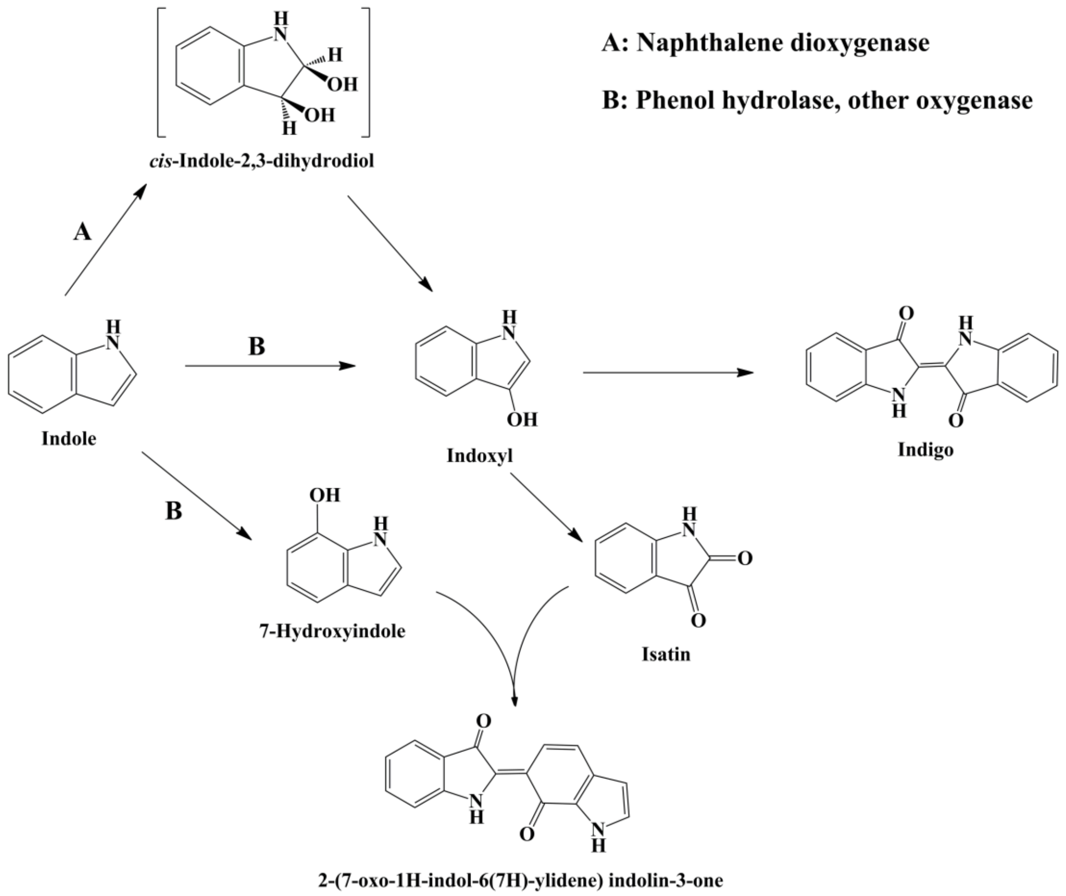

Fig C. Proposed pathways for the production of indigoids from indole by the activated sludge systems. Indole can be hydroxylated to form indoxyl by oxygenases A and B, which may be further oxidized to form isatin. Condensation of two molecules of indoxyl by air oxidation will lead to the production of indigo. Besides, indole can be also oxidized by oxygenase B to form 7-hydroxyindole, which can react with isatin to form 2-(7-oxo-1H-indol-6(7H)-ylidene) indolin-3-one. Oxygenase A may be naphthalene dioxygenase in G1; B may be phenol hydrolase in G2 or other oxygeases in G3.

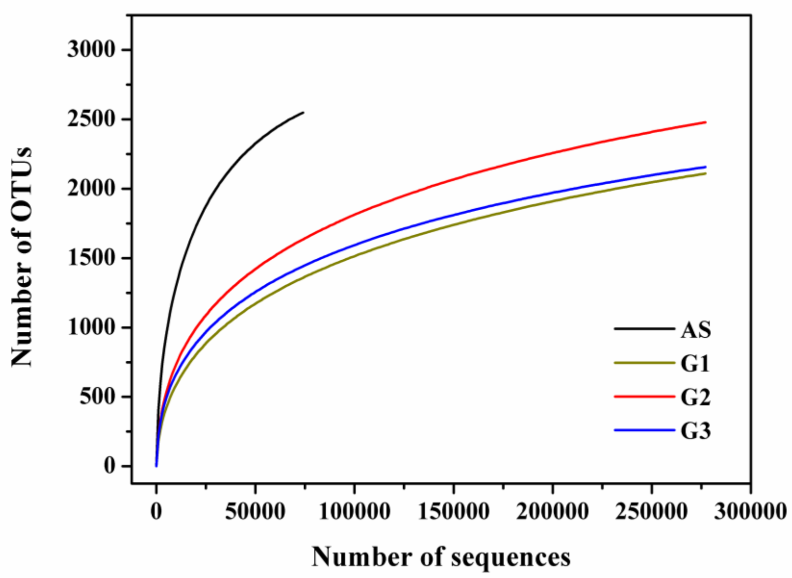

Fig D. Rarefaction curves base on Illumina MiSeq sequencing of microbial communities. The OTUs were defined by 3% distances. The rarefaction curve of AS was derived from 3 samples, while those of each group were from 11 samples. The detailed group setup was presented in Table A in S1 File.

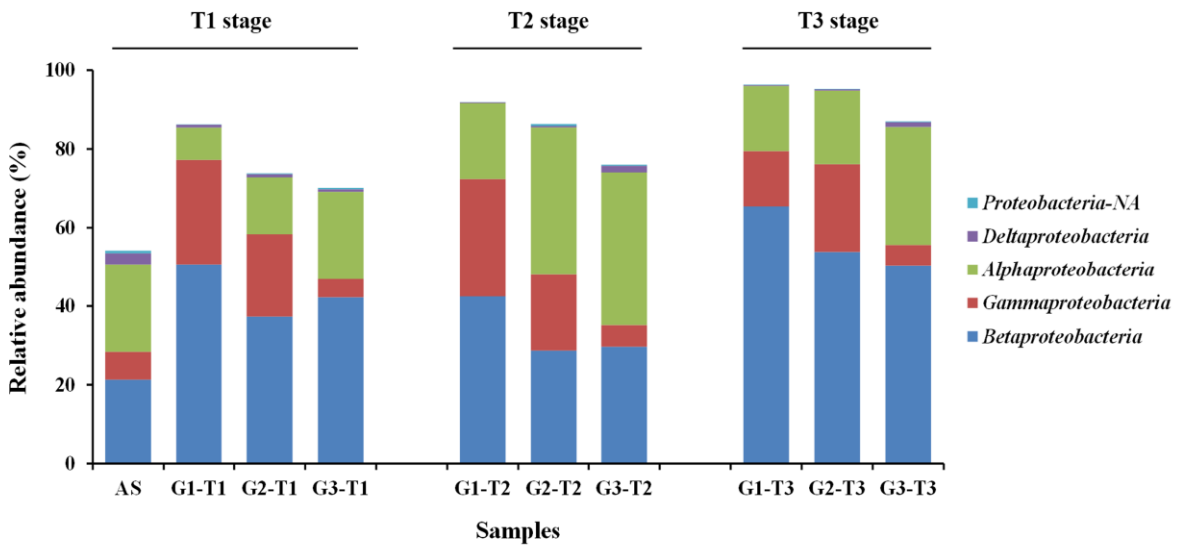

Fig E. Relative abundance of *Proteobacteria* subdivisions in each group at different stages.
